# Formulation Development and Ex-Vivo Permeability of Curcumin Hydrogels under the Influence of Natural Chemical Enhancers

**DOI:** 10.3390/gels8060384

**Published:** 2022-06-16

**Authors:** Asif Nawaz, Arshad Farid, Muhammad Safdar, Muhammad Shahid Latif, Shakira Ghazanfar, Nosheen Akhtar, Soad K. Al Jaouni, Samy Selim, Muhammad Waseem Khan

**Affiliations:** 1 Advanced Drug Delivery Lab, Gomal Centre of Pharmaceutical Sciences, Faculty of Pharmacy, Gomal University, Dera Ismail Khan 29050, Pakistan; safdarlaghari08@yahoo.com (M.S.); shahidlatif1710@gmail.com (M.S.L.); 2 Gomal Center of Biochemistry and Biotechnology, Gomal University, Dera Ismail Khan 29050, Pakistan; 3 National Institute for Genomics Advanced Biotechnology, National Agricultural Research Centre, Park Road, Islamabad 45500, Pakistan; shakira_akmal@yahoo.com; 4 Department of Biological Sciences, National University of Medical Sciences, Islamabad 45500, Pakistan; nosheenakhtar@numspak.edu.pk; 5 Department of Hematology/Oncology, Yousef Abdulatif Jameel Scientific Chair of Prophetic Medicine Application, Faculty of Medicine, King Abdulaziz University, Jeddah 21589, Saudi Arabia; saljaouni@kau.edu.sa; 6 Department of Clinical Laboratory Sciences, College of Applied Medical Sciences, Jouf University, Sakaka 72388, Saudi Arabia; sabdulsalam@ju.edu.sa; 7 Institute of Pharmaceutical Sciences, Khyber Medical University, Peshawar 25000, Pakistan; khanwaseem6065@gmail.com

**Keywords:** topical, hydrogels, permeation enhancers, curcumin, essential oils

## Abstract

Background: The aim of the present research was to formulate and evaluate curcumin hydrogel and to investigate the potential of natural essential oils as permeation enhancers. Methods: Curcumin 2% *w*/*w* hydrogel containing various concentrations of eucalyptus oil, aloe vera oil and clove oil was developed using carboxy methyl cellulose (CMC) as a gelling agent. Differential scanning calorimetry and Fourier Transform infrared spectroscopy were used to evaluate the compatibility between the drug and the excipients. In order to assess the efficacy of the formulation; rheological properties, skin irritation studies, in vitro release, ex vivo permeation and retention studies were conducted. Results: DSC and FTIR suggest no in-compatibility between curcumin and excipients. Studies proved that addition of suitable natural permeation enhancers to the hydrogels improved the in vitro release and ex vivo permeation and retention of curcumin. From the various natural essential oils, the aloe vera oil at a concentration of 3% *w*/*w* had the greatest effect on the permeability rate and skin retention of the Curcumin and produces the highest enhancement ratio amongst all the concentrations of essential oils examined. Conclusion: Aloe vera oil enhances the permeation of curcumin across the skin by altering the complex structure of the stratum corneum without itself undergoing any change. The developed curcumin hydrogels along with natural essential oils may present an effective choice regarding skin infection/wound healing.

## 1. Introduction

Drug delivery to or via skin is an effective route for local and systemic administration of therapeutically active agents. However, skin and in particular the stratum corneum (upper most layer of skin) provides resistance to drug absorption [1]. In order to permeate and absorb sufficient amount of drug for therapeutic effects, permeation should be enhanced. The primary or basic approach to overcome skin resistance to drug permeation; is the skillful selection of penetration enhancers [2]. Permeation enhancers are substances that facilitate penetration by reversibly altering the organized/complex structure of the skin. The effects of penetration enhancers on the skin should be reversible, non-allergenic, non-toxic, compatible with drugs and excipients and non-irritating. Most of the chemicals that are used as penetration enhancers possess localized and systemic side effects. Therefore, an investigation into possible penetration enhancers that are safe, effective and cost-effective is desirable.

Various natural oils have been increasingly used as permeation enhancers due to their excellent safety profile [3]. Recent studies on natural essential oils show that these oils increase skin penetration in transdermal applications. Essential oils increase skin penetration by interacting with the stratum corneum. They were found to be successful in increasing skin penetration of both lipophilic and hydrophilic drugs. Moreover, essential oils do not accumulate in the body since they are volatile, and also are easily discharged from the body through feces and urine. They are preferred because essential oils are natural, mostly do not damage the skin while increasing skin penetration, less toxic, and less allergenic [4].

Topical drug delivery is not suitable/justifiable for all types of drugs. Curcumin is used as the drug of choice in the present study. Curcumin is a major naturally occurring, low-molecular-weight (368.3 g/mol) polyphenolic, hydrophobic compound with a logP value of three [5]. Curcumin is obtained from the plant curcuma longa and is widely used as a spice, and or yellow coloring agent in food. Curcumin possesses many biological activities such as strong anti-inflammatory, antioxidant, antimicrobial, anti-parasitic, anti-diabetic, anti-fungal effects and even anti-cancer activity [6,7]. Curcumin has low bioavailability due to poor/low absorption, low gastro intestinal dissolution rate, rapid metabolism and rapid systemic elimination. These effects may be overcome by topical administration of curcumin [5,8]. Various topical formulations of curcumin were studied, which includes nanoemulsion, nanoparticles, lipid-based self-emulsifying systems, ethosomes and hydrogels [9]. Among these formulations hydrogels were easily prepared, easy to apply on skin and easily washable. The permeability of drugs from hydrogels were comparatively low as compared to other formulations. In order to increase the drug permeability across skin, various natural penetration enhancers were used. Carboxy methylcellulose (CMC) was used as a gelling agent in the present study. CMC also acts as a permeation enhancer [10]. The combination of CMC and natural oils in hydrogels formulation may result in higher permeability and skin drug retention.

In the present study, attempts have been made to design, formulate and evaluate topically applied hydrogel containing curcumin and to explore the enhancement effect of the natural essential oils.

## 2. Materials and Methods

### 2.1. Materials

Curcumin (RMY Exporter, Mumbai, India) was used as the drug of interest. Eucalyptus oil, aloe vera oil and clove oil (Hamdard, Islamabad, Pakistan) were used as chemical enhancers. Carboxy methylcellulose (CMC) (Sigma Aldrich, Steinheim am Albuch, Germany), was used as gelling polymer. Ethanol, Sodium Hydroxide, Potassium dihydrogenphosphate, Triethanolamine (TEA) were purchased from Merck, Germany. Formalin (Sigma Aldrich, Steinheim am Albuch, Germany) was used as standard irritant. All the chemicals were used without further purification.

### 2.2. Differential Scanning Calorimetric Analysis (DSC)

The physicochemical compatibility of the drug and the used excipients were tested by differential scanning calorimetric (DSC) analysis. DSC thermograms of the drug alone and drug-excipients physical mixture (CPM) were derived from a DSC (Perkin Elmer, Shelton, CT, USA) with a thermal analysis data station system, computer and a plotter interface. The instrument was calibrated with an indium standard. The samples (3 mg) were heated (0–300 °C) at a constant scanning speed (10 °C/min) in sealed aluminum pans using nitrogen as purging gas [11].

### 2.3. ATR-FTIR Analysis

ATR-FTIR spectra of Curcumin, CMC, essential oils, and the hydrogel were examined using the ATR-FTIR spectrometer (PerkinElmer, MA, USA). The samples were placed on sample holder and scanned between 4000–400 cm^−1^.

### 2.4. Method for Preparation of Hydrogel

Carboxy methyl cellulose (CMC) was accurately weighed (2 g) and dispersed in distilled water and stirred continuously at 300 rpm for 2 h. The weighed quantity of Curcumin (2%) was dispersed in ethanol and then added to the carboxy methyl cellulose mixture with continuous stirring until homogenous dispersion was achieved. To this dispersion different concentrations of natural chemical enhancers were added and mixed for 1 h. The dispersion was then neutralized and made viscous by the addition of triethanolamine (TEA) to obtain the hydrogel [12]. The hydrogel was labeled and stored under controlled conditions of temperature in an incubator until further use. The compositions of different hydrogel formulations are listed in Table 1.

### 2.5. Physicochemical Evaluation of Hydrogels

#### 2.5.1. pH of Hydrogel

The pH levels of the various hydrogel formulations were determined, using a digital pH meter (Denver, CO, USA). The determination was carried out in triplicate and average of the three readings was recorded.

#### 2.5.2. Drug Content

A specific quantity (100 mg) of the developed hydrogel was dissolved in 100 mL of ethanol. The volumetric flask containing hydrogel solution was shaken for 2 h on mechanical shaker in order to achieve complete solubility of drug. This solution was then filtered through a membrane filter (pore size 0.45 mm). The absorbance of the sample was determined on UV–Visible spectrophotometer at wavelength of 425 nm using ethanol as a blank. The UV method for curcumin analysis was validated for various validation parameters. The linearity range was found to be 3–28 µg/mL with an R^2^ (coefficient of correlation) value of 0.9987. The LOD and LOQ were found to be 0.5 and 1.3 µg/mL, respectively. The robustness was found to be 99.1%. The concentration of Curcumin was estimated from the regression equation of the calibration curve [12].

#### 2.5.3. Homogeneity

All the developed hydrogels were tested for homogeneity by visual inspection. They were tested for their appearance and feel on application.

#### 2.5.4. Viscosity

The viscosity of formulated hydrogels was measured by Brookfield viscometer (AMETEK Brookfield, Middleboro, MA, USA). The spindle (#4) of viscometer was used and rotated at 2.5, 4, 5 and 10 rpm. Samples were measured at 30 °C. Three readings were taken and averaged.

#### 2.5.5. Acute Skin Irritation Test

The skin irritation test was conducted on healthy male Sprague Dawley rats. The procedure was approved from Ethical Review Board of Gomal University, Pakistan. Dorsal area of the rats were shaved with a razor and marked as a circular area (0.77 cm^2^) with a felt-tip marker. The animals were divided into 3 groups (*n* = 5) and treated as follows.

Group I, served as Non-treated (Normal); Group II served as Control (Applied with optimized Curcumin hydrogel) and Group III served as Formalin (A standard irritant; 0.8% *v*/*v*).

The animals were treated twice daily with new hydrogel/formalin solution up to 7 days and finally the treated skin was examined visually for erythema and edema. The skin irritation (erythema and edema) was evaluated by visual scoring using a modified method of [13].

### 2.6. In Vitro Release Study

In vitro release study of formulated hydrogel was carried out using Franz Diffusion cell with effective diffusional surface area of 0.77 cm^2^ and a receptor cell volume of 5 mL. The receptor compartment was filled with dissolution medium consisting of phosphate buffer pH 5.5 and 1.5% polysorbate 80, and temperature was maintained at 32 °C (in-simulation to skin pH and temperature) [14]. The cellulose acetate membrane (Sigma Aldrich, Germany) with a pore size of 0.45 µm was fixed between the donor and receptor compartment. The donor compartment was charged with 1 g of hydrogel. A 2 mL volume of sample was collected from the receptor compartment at predetermined time intervals, i.e., 0, 0.5, 1, 2, 4, 8, 12, 16, 20 and 24 h. The amount of Curcumin in the samples was analysed using a UV–Visible spectrophotometer (Shimadzu 1601, Kyoto, Japan) at a wavelength of 425 nm. The experiment was performed in triplicates and the results were averaged.

### 2.7. Ex Vivo Permeation Study

Rats within a weight range of 200–250 g were decapitated. The abdominal hair of the rats were shaved using an electric razor after sacrificing with cervical dislocation method. The abdominal skin was surgically removed and adhering subcutaneous fat was carefully cleaned. The skin was thoroughly washed with normal saline, dried, wrapped in aluminum foil and stored in freezer at −20 °C until further use.

The in vitro drug permeation from the hydrogel formulations was studied across excised rat skin using Franz Diffusion Cell. The receptor compartment was filled with dissolution medium consisting of phosphate buffered saline pH 7.4 and 1.5% polysorbate 80 (in-simulation to blood pH). The rat skin was fixed between the donor and receptor compartment of Franz cell in such a way that the epidermis was exposed to open air, while the dermis faced the receptor compartment. The donor compartment was charged with 1 g of the sample hydrogel and covered with a piece of aluminum foil to prevent drying out. The temperature of the cell was maintained at 37 °C by surrounding water in jacket and the medium was stirred by magnetic stirrer at 100 rpm. The samples were collected from the receptor compartment at predetermined intervals, i.e., 0, 0.5, 1, 2, 4, 8, 12, 16, 20 and 24 h, and replaced with equal volume of fresh receptor solution to keep the volume constant. The amount of Curcumin in the samples was analysed using a UV–Visible spectrophotometer (Shimadzu 1601, Kyoto, Japan) at wavelength 425 nm. The UV method was validated for various validation parameters to avoid the possible interferences of other substances.

### 2.8. Skin Drug Retention

The skin after the permeation experiment was carefully removed and cut into small pieces. The pieces of skin were homogenized using a tissue homogenizer and drug present in the skin was extracted in 20 mL of methanol under sonication. The extract was filtered using membrane filter 0.45 micron and analyzed on UV at 425 nm.

### 2.9. Statistical Analysis

The cumulative amount permeated was evaluated statistically using a one-way analysis of variance (ANOVA). Difference between the cumulative amounts from the formulations were considered statistically significant at a *p*-value ≤ 0.05.

## 3. Results and Discussion

The aim of the present research work was to evaluate the skin penetration ability of essential oils and their potential application for curcumin skin delivery. Carboxy methyl cellulose-based curcumin hydrogels were prepared and evaluated physicochemically.

### 3.1. Differential Scanning Calorimetric Analysis (DSC)

**Figure 1:**
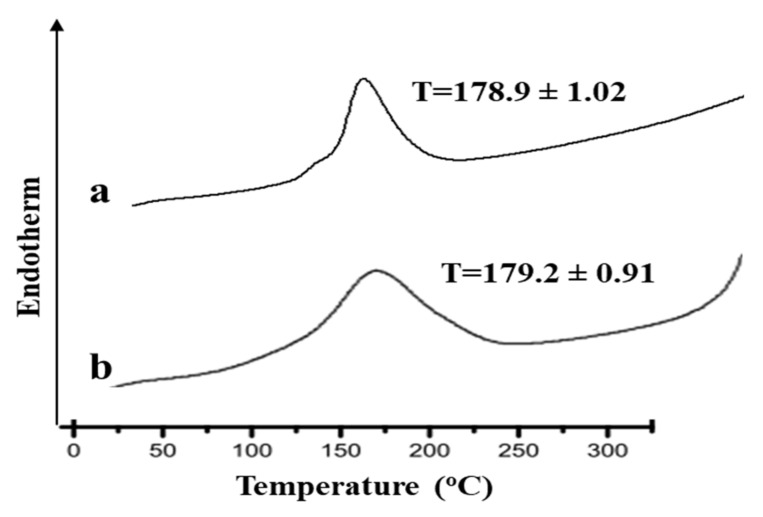
DSC thermograms of (**a**) pure drug and (**b**) physical mixture of drug and polymer.

Figure 1 shows the thermograms of the pure drug (Curcumin) and their physical mixture (drug and Polymer (CMC)). The DSC analysis of the pure drug alone elicited an endothermic peak at 178.9 °C, which corresponds to the melting point of the drug in the crystalline form [15]. In the DSC thermogram of physical mixture of drug and polymer, the characteristics peaks of the drug were observed. Thus, it was thought that there was no conclusive evidence of interaction between the drug and the excipients.

### 3.2. FTIR Analysis

**Figure 2:**
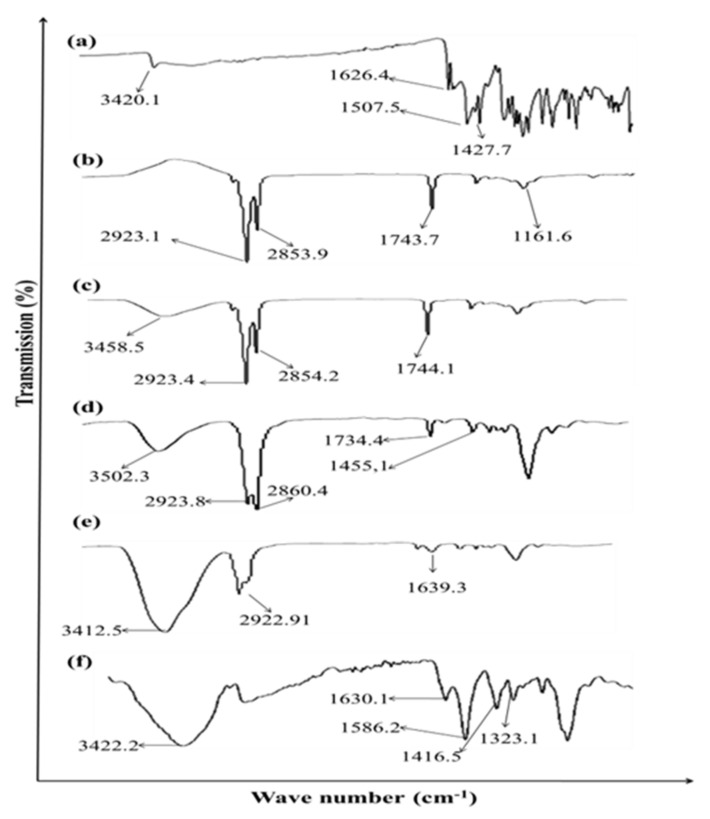
ATR-FTIR spectra of (**a**) Curcumin, (**b**) Eucalyptus oil, (**c**) Aloe vera oil, (**d**) Clove oil, (**e**) CMC and (**f**) Hydrogel.

Figure 2 demonstrates the FTIR spectra of Curcumin, eucalyptus oil, aloe vera oil, clove oil, CMC and hydrogels. The characteristic peaks of curcumin are observed at 3420.1 cm^−1^ due to the phenolic O-H stretching vibration, CH_2_ stretching vibration at 2926 cm^−1^, and bands at 1639 cm^−1^ assigned to the response of C=O (ketone) stretching vibration. C=O and C=C characteristic vibrations are observed at 1521 cm^−1^, enolic C-OH bending vibration is detected at 1423 cm^−1^, C-O stretching vibrations are detected at 1241 cm^−1^ while at 1021 cm^−1^ the C-O-C stretching vibration signature is observed. The FTIR spectrum of the Hydrogels loaded with curcumin showed the characteristics peaks of both Curcumin, polymer and other excipients with a minor shifting and/or decreased intensity. These findings demonstrated the absence of interactions between Curcumin and different excipients. The shifting of the peak at 1423 cm^−1^ to 1416 cm^−1^ in hydrogels indicates electrostatic interaction and or Hydrogen bonding [9]. The reduced intensity and minor shifting of the characteristic peaks of Curcumin specially -OH (3420 cm^−1^) and -NH (1626 cm^−1^) might be attributed to the presence of some bonds, such as van der Waals forces, hydrogen bonds, or dipole interactions, between Curcumin and other excipients that results in increasing the entrapment of Curcumin within hydrogels.

### 3.3. Physicochemical Evaluation of Hydrogel

Different hydrogel formulations containing 2% Curcumin with or without varying concentrations of natural chemical enhancers were prepared (Table 1) and evaluated for pH, drug content, viscosity, homogeneity, skin irritation and in vitro drug release and permeation across the rat skin.

As shown in Table 2, the pH values of all developed hydrogels range from 6.0 to 7.2, which lies within the normal pH range and would not produce any skin irritation. The pH of formulations plays an important role in topical delivery. If pH of the topical formulation is 4 or lower than 4, it can result in skin irritation [16]. The drug content of all formulation ranges from 86 to 94.1%, which shows content uniformity. All developed hydrogels showed good homogeneity with absence of lumps, as no particles were seen in the smear of hydrogel under the microscope. All the preparations were clear. Spreadability of the formulated hydrogels were in the range from 6.1 to 6.8 g cm/s, which is indicative of good spreadability of hydrogels. The values of spreadability indicate that hydrogel is easily spreadable by applying a small amount of force [17].

The viscosity of a topical hydrogel is one of the important physical parameters which is always inversely proportional to the extent of permeation if diffusion through vehicle is the rate-limiting step. In general, an increase in viscosity of vehicles would cause a more rigid structure and decrease the drug release rate [18]. The viscosity of different formulations ranges from 15,645 to 17,760 cps at 10 rpm (Table 2). The viscosity of hydrogel reduces with the addition of essential oils. The skin irritation studies of developed hydrogels were carried out on rats and confirmed the absence of any erythema and edema on the applied surface after 12 h of application as shown in Table 2. This suggested that the hydrogel formulation exhibits no irritation thus, improving skin acceptability and patient suitability and was found to be safe for topical application.

### 3.4. In Vitro Release Study

**Figure 3:**
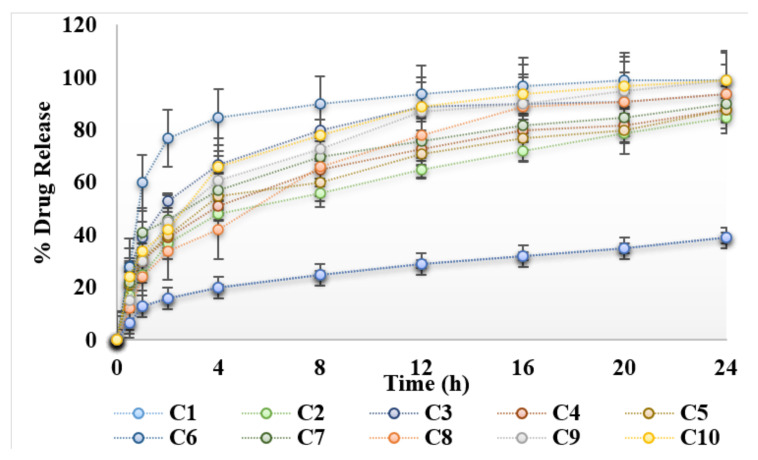
Release profile of hydrogels under the influence of essential oils.

In vitro release studies were conducted using Franz diffusion cell across synthetic/cellulose membrane. Results of release studies are shown in Figure 3. Initially there was a burst release of curcumin, followed by sustained release behavior. Drug located on the surface of hydrogels and not properly incorporated into hydrogels, results in initial burst release. Carboxy methylcellulose is a hydrophilic polymer, it swells in a hydrophilic environment and helps to sustain the release of drug [19]. It was found that the lowest percentage of drug was released from the hydrogels formulated without natural enhancers. Incorporation of natural chemical enhancers results in increased release of the drug. Natural oils help release of the drug from the polymer matrix by modifying/loosening the polymer matrix/network. Maximum release of curcumin was obtained with 3% aloe vera oil (Figure 3). The release kinetics of the hydrogels were determined using Korsmeyer–Peppas model. The release profiles from the prepared hydrogels were fitted in the Korsmeyer–Peppas model in order to obtain the *n* value. The *n* values were in the range of *n* > 1, indicating that the drug release from the hydrogels follow a non-Fickian (anomalous) mechanism.

### 3.5. Ex Vivo Permeation Study

**Figure 4:**
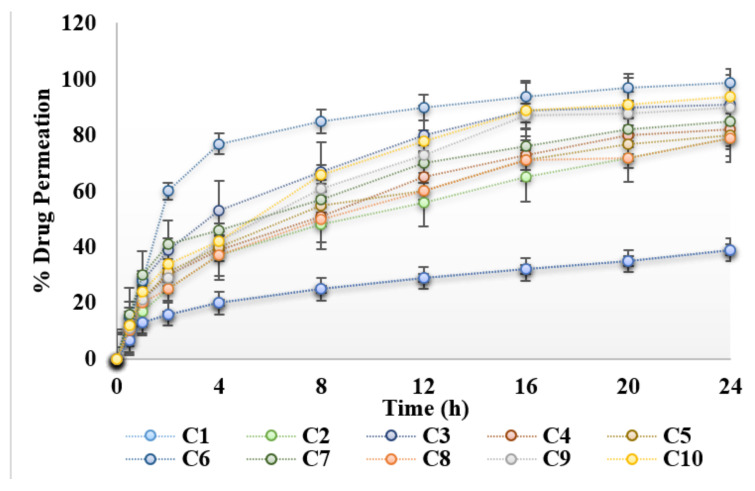
Permeation profile of hydrogels under the influence of essential oils.

Ex vivo drug permeation study across rats’ skin is shown in Figure 4. The permeation of curcumin across skin was low in the absence of natural chemical enhancers. Carboxy methylcellulose is a hydrophilic polymer and also acts as penetration enhancer. The results obtained, indicate that the presence of natural chemical enhancers results in increased permeation of the drug across the skin (Figure 4). Essential oils are mixtures of complex lipids, which interact with the skin resulting in increased permeation [19]. The presence of 3% aloe vera oil seems to have the greatest effect on the permeation of curcumin across the rats’ skin. In fact, the permeability rate of curcumin hydrogel containing 3% aloe vera oil is significantly (ANOVA, *p* < 0.05) greater than the other concentrations studied (Figure 4). Essential oils reversibly alter the lipid structure of the stratum corneum which ultimately results in pore formation in the skin. Hence, this results in an increase in permeation of drug across the skin. Aloe vera oil contains fatty acids, which enhances skin permeability by altering/disordering the organized alkyl chains of phospholipids, resulting in lipid fluidization [3]. Lipid fluidization results in the increase permeation of the drug across the skin. Previously, it was reported that aloe vera oil extract enhances permeation of the lipid bilayers in the stratum corneum which reduces the barrier resistance of stratum corneum, and increase intracellular transport by dekeratinization of corneocytes [20]. Essential oils contain fatty acids. Fatty acids bearing a C18 chain and double bond, form “link” structures and have the potential to disrupt the organized skin lipids [21]. Apparently, permeability of curcumin increases up to 3% of aloe vera oil followed by the observation of a slight decrease when the concentration of aloe vera oil increases (Figure 4). The presence of a large amount of aloe vera oil could slow down the partitioning of curcumin out of the base-hydrogel to the stratum corneum and hence results in the decrease in permeation of curcumin across the rat skin. Clove oil and eucalyptus oil also increases the permeation of curcumin across the skin as compared to control hydrogel (C1). However, the percent permeation was lower than the hydrogels containing aloe vera oil. Similarly, a significant increase in flux (83.12 ± 1.37 μg/h/cm^2^) of C6 was observed when compared with control hydrogel C1 (33.41 ± 1.69 μg/h/cm^2^).

### 3.6. Skin Drug Retention

**Figure 5:**
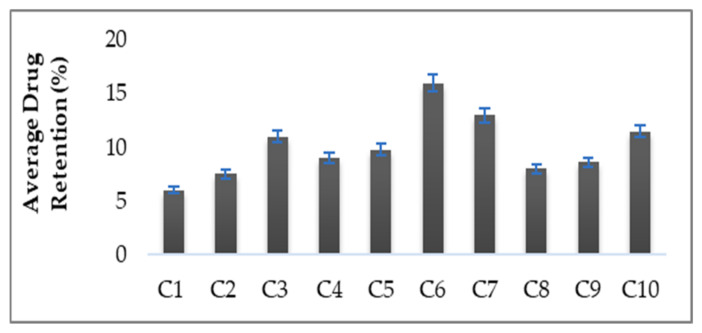
Percent Skin Drug Retention.

The skin drug retention study is an important parameter for local infection/targeting. It was found that, after 24 h, 16 ± 1.02% of the drug was retained in the skin from hydrogel C6 as compared to 6  ±  0.59% from the control hydrogel (Figure 5). Thus, the amount of drug retained in the skin for hydrogel (C6) was found to be higher than the control hydrogel. This dermal retention of curcumin was attributed to increased contact with corneocytes, extraction of lipids of stratum corneum and the sustained release properties of hydrogels. The presence of essential oils in the hydrogels helps in the permeation and retention of curcumin in the skin by reversibly altering the complex structure of the skin. It was concluded that the enhanced curcumin retention in the skin is mainly attributed to the presence of essential oils in the hydrogel formulations.

## 4. Conclusions

Curcumin hydrogels were prepared using carboxy methyl cellulose as base polymer and essential oils as permeation enhancers. All the prepared formulations were physicochemically evaluated. The prepared formulations were stable and demonstrated no skin irritation effect. The addition of essential oils enhances the in vitro release and ex-vivo permeation of the curcumin. Among all the essential oils, aloe vera oil at a concentration of 3% *w*/*w* had the greatest effect on the penetration of curcumin across the rat skin. Aloe vera oil alters the complex structure of phospholipids (fluidizes the lipids of the stratum corneum) which results in increased permeation of curcumin across the rat skin. The developed curcumin hydrogels along with essential oils may present an effective choice for skin infection/wound healing.

## Figures and Tables

**Table 1:** Composition of Curcumin hydrogels.

F. Code	Drug (g)	Polymer (CMC) (g)	TEA (mL)	Ethanol (mL)	Eucalyptus Oil (%)	Aloe Vera Oil (%)	Clove Oil (%)	Water QS to Make 100 g
**C1**	2	2	1	10	-	-	-	100
**C2**	2	2	1	10	1			100
**C3**	2	2	1	10	3			100
**C4**	2	2	1	10	5			100
**C5**	2	2	1	10		1		100
**C6**	2	2	1	10		3		100
**C7**	2	2	1	10		5		100
**C8**	2	2	1	10			1	100
**C9**	2	2	1	10			3	100
**C10**	2	2	1	10			5	100

**Table 2:** Physicochemical Evaluation of the hydrogel.

F. Code	pH	% Drug Content	Homogeneity	Viscosity (cps) at 10 rpm	Skin Irritation Test
**C1**	6.0 ± 0.6	86.1 ± 1.3%	+	17,760 ± 4.3	Nil
**C2**	7.1 ± 0.4	91.3 ± 1.7%	+++	16,002 ± 5.2	Nil
**C3**	6.4 ± 0.7	88.9 ± 2.1%	++	16,230 ± 4.8	Nil
**C4**	6.8 ± 0.3	93.7 ± 2.8%	+++	16,432 ± 5.1	Nil
**C5**	7.0 ± 0.2	92.9 ± 1.9%	+++	16,421 ± 4.9	Nil
**C6**	7.1 ± 0.2	94.6 ± 2.2%	++	15,645 ± 5.4	Nil
**C7**	6.3 ± 0.5	91.2 ± 2.7%	++	15,832 ± 3.9	Nil
**C8**	6.7 ± 0.6	92.6 ± 1.8%	+++	15,991 ± 4.3	Nil
**C9**	6.8 ± 0.7	93.8 ± 2.1%	++	16,121 ± 4.9	Nil
**C10**	6.5 ± 0.9	88.8 ± 2.5%	+	16,532 ± 5.8	Nil

+++ Excellent, ++ Good, + Satisfactory.

## Data Availability

Not applicable.
